# A maize heat shock factor *ZmHsf11* negatively regulates heat stress tolerance in transgenic plants

**DOI:** 10.1186/s12870-022-03789-1

**Published:** 2022-08-20

**Authors:** Qianqian Qin, Yujun Zhao, Jiajun Zhang, Li Chen, Weina Si, Haiyang Jiang

**Affiliations:** National Engineering Laboratory of Crop Stress Resistance Breeding, School of Life Sciences, Anhui Agricultural University, Hefei, 230036 China

**Keywords:** Maize, ZmHsf11, Heat shock factor, Heat stress, Oxidative stress

## Abstract

**Background:**

Heat shock transcription factors (Hsfs) are highly conserved among eukaryote and always play vital role in plant stress responses. Whereas, function and mechanism of Hsfs in maize are limited.

**Results:**

In this study, an HSF gene *ZmHsf11*, a member of class B Hsfs, was cloned from maize, and it was up-regulated under heat treatment. ZmHsf11 was a nuclear protein with no transcriptional autoactivation activity in yeast. Overexpression of *ZmHsf11* gene in *Arabidopsis* and rice significantly reduced the survival rate under heat shock treatment and decreased ABA sensitivity of transgenic plants. Under heat stress, transgenic rice accumulated more H_2_O_2_, increased cell death, and decreased proline content compared with wild type. In addition, RT-qPCR analysis revealed that *ZmHsf11* negatively regulated some oxidative stress-related genes *APX2, DREB2A, HsfA2e, NTL3, GR* and *HSP17* under heat stress treatment.

**Conclusions:**

Our results indicate that *ZmHsf11* decreases plant tolerance to heat stress by negatively regulating the expression of oxidative stress-related genes, increasing ROS levels and decreasing proline content. It is a negative regulator involved in high temperature stress response.

**Supplementary Information:**

The online version contains supplementary material available at 10.1186/s12870-022-03789-1.

## Background

As sessile organisms, plants are generally exposed to adverse environments, and high temperature stress has a great impact on their growth, development and reproduction [1]. Under high temperature stress, plants can sense temperature changes and activate cellular signaling and metabolic pathways to adapt to new environmental conditions [2]. This involves the expression of some genes or proteins, including phytohormone or signal transduction-related genes, Ca^2+^ signaling pathways, reactive oxygen species-related genes, heat shock transcription factors (Hsfs) and heat shock proteins [3–7]. Heat shock transcription factors (Hsfs) play a major role as regulators in the heat stress regulation pathway [8].

Heat shock transcription factors (Hsfs), as major transactivating element in the heat stress signaling pathway, activate the expression of related genes under heat shock and regulate the process of heat stress response in plants [9]. Hsfs were classified into classes A, B and C based on the structural features of DNA binding domain (DBD) and oligomerization domain (OD) and the presence of class-specific motifs [10]. *HsfA* genes play a key role in heat stress response, and their members can be roughly divided into A1-A9 [11]. In *Arabidopsis*, four *HsfA1s* (a/b/d/e) are functionally redundant, and their *hsfa1a/b/d* triple and *hsfa1a/b/d/e* quadruple mutants had significantly reduced survival after exposure to HS. Expression of HS-responsive genes, including molecular chaperones and transcription factors, was also impaired, and tolerance to HS stress was greatly in both the triple and quadruple mutants [12]. A temperature-dependent repression (TDR) domain of *AtHsfA1d* represses the transactivation activity of *HsfA1d* through interaction with *HSP70* and *HSP90*, overexpression of *HsfA1d* lacking the TDR domain induces the expression of heat shock proteins and thereby enhancing the high temperature tolerance of *Arabidopsis* [13]. Overexpression and small RNA interference of *HsfA1* in tomato elucidated its heat shock function in vivo [14]. *HsfA2* as a regulatory amplifier for a subset of genes in heat response, increasing the expression of the heat responsive genes *APX2* and *HSP* genes, and enhancing the acquired thermotolerance in *Arabidopsis* [15–17]. In tomato, *HsfA2* is an essential coactivator of *HsfA1a*, and maintains pollen thermotolerance by controlling a number of genes with important developmental functions [18]. The sustained accumulation of histone H3 lysine 4 (H3K4) methylation was depended on *HSFA2* and highly induced the expression of related genes after heat shock, and the heteromeric complexes containing both *HSFA2* and *HSFA3* potently promoted transcriptional memory through positively affecting H3K4 hypermethylation [17, 19]. In *Arabidopsis*, *HsfA3* was a heat shock-inducible gene under the regulation of *DREB2A*, and *DREB2A* itself was an HS-inducible gene that played a major role in both heat shock and lack of water stress responses [20, 21]. In tomato, *HsfA3* can interact with MAP kinase to modulate the response to heat stress [22]. Overexpression of lily *HsfA3s* in *Arabidopsis* increased temperature tolerance and salt sensitivity through altered proline catabolism [23]. *LlHsfA4* enhanced basal thermotolerance of lily by regulating ROS metabolism [24]. Whereas, *HsfA5* specifically interfered with the oligomeric state of *HsfA4*, thereby interfering with its DNA-binding ability [25]. Overexpression of *TaHsfA6b* enhanced the heat tolerance of crops through altering the expression of some stress-related genes [26]. *AtHsfA8* acted as a redox-sensing transcription factor in stress-responsive transcriptional networks, and *AtHsfA9* was regulated by the seed-specific transcription factor *ABI3* and was expressed only at later stages of seed development [27–29].

Compared with Class A Hsfs, the function of Class B Hsfs is less clear. Class B Hsfs are significantly different from class A in protein structure, including B1-B4. They do not contain nuclear export signaling and activation domains and thus do not have transcriptional activation activity per se [30]. In *Arabidopsis*, *HsfB1* and *HsfB2b* were found to act as inhibitors of HSR genes in non-stressed cells and during stress recovery, but were necessary for heat stress-induced heat shock protein gene expression under heat stress, *hsfb1-hsfb2b* knockout mutant exhibited higher basal thermotolerance than wild-type [31, 32]. *HsfB1* was a core regulator of the heat stress response and played a dual role as a transcriptional co-activator and general repressor of *HsfA1a* in tomato [33]. *HsfB2b* responded to abiotic stress responses of the circadian clock through inhibition of *PRR7* [34]. While *HsfB3* and *HsfB4* had only been reported on cell death in *Arabidopsis* [35]. Class C Hsfs include C1 and C2. There are few studies on HsfC, such as the overexpression of *FaHsfC1b* gene improved the heat tolerance of *Arabidopsis* [36].

Maize, as one of the food and feed crops for agricultural planting, is beneficial to its high yield and high quality under a suitable growing environment [37, 38]. Therefore, it is especially important to study the mechanism of high temperature resistance in maize. The *Hsf* gene family played an important regulatory role in the response to high temperature stress, but there are few studies on the function of the maize *HsfB* gene, only the enhanced sensitivity of *ZmHsfB1* to salt and drought has been reported [39]. In this study, a member of class B Hsfs from maize known as *ZmHsf11* was cloned, and identified its function in response to high temperature stress. We found that high temperature induced the up-regulated expression of *ZmHsf11*, the protein was localized in the nucleus and had no transcriptional autoactivation activity. Notably, overexpression of *ZmHsf11* in *Arabidopsis* and rice significantly increased the high temperature stress sensitivity and decreased ABA sensitivity of transgenic plants, decreased the expression of oxidative stress-related genes, reduced proline content, and increased H_2_O_2_ content in rice, suggesting that *ZmHsf11* negatively regulated plant high temperature tolerance. 

## Results

### Identification and sequence analysis of *ZmHsf11*

**Fig. 1:**
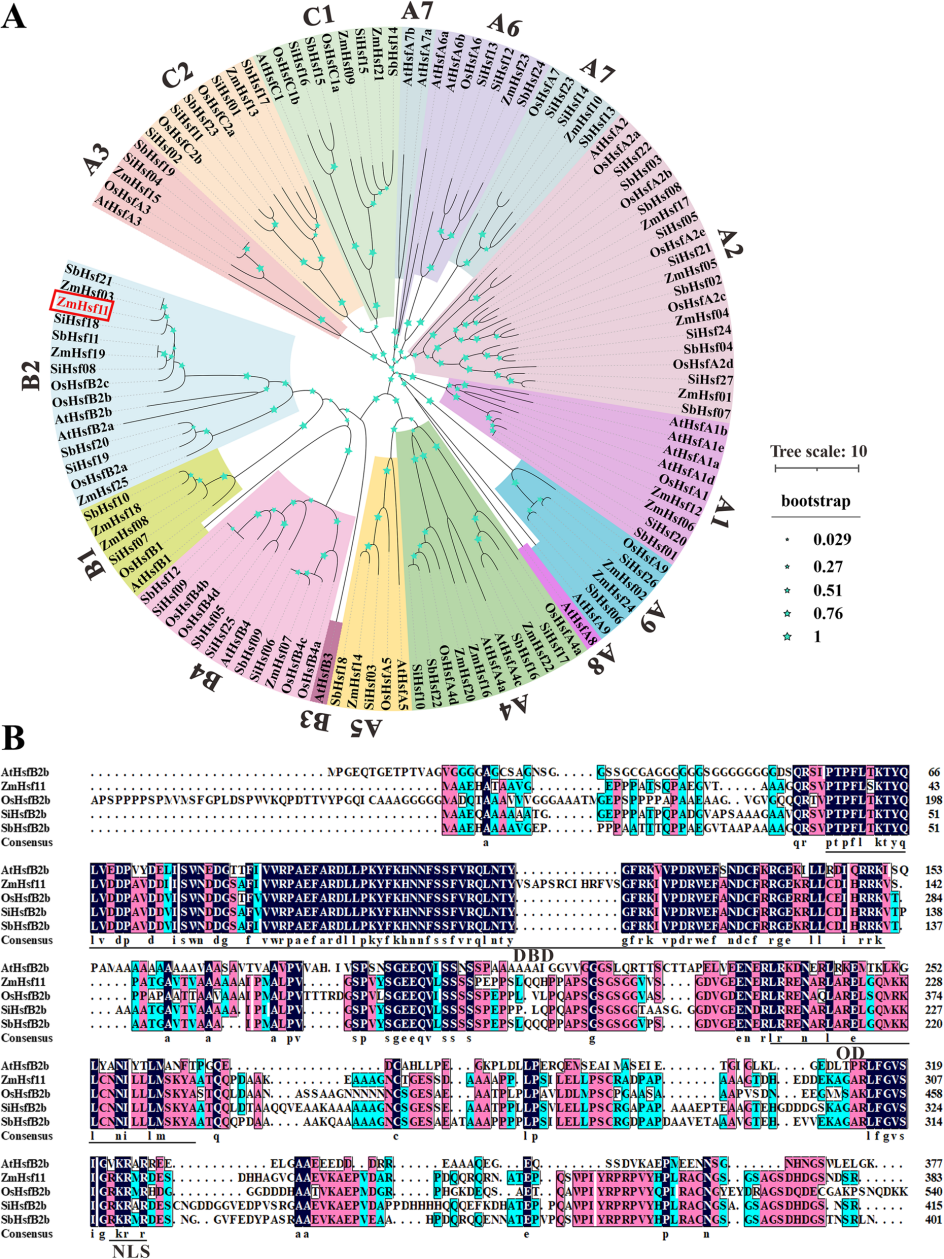
Sequence and phylogenetic analyses of *ZmHsf11*. **A** Phylogenetic analysis of *Hsf* gene families in *Arabidopsis*, rice, sorghum, millet and maize. **B** Alignment of the amino acid sequence of ZmHsf11 with its homologous proteins in other species. The related protein names are as follows: *ZmHsf11*, GRMZM2G098696, from *Zea mays*; *AtHsfB2b*, NC_003075.7, from *Arabidopsis thaliana*; *OsHsfB2b*, LOC_Os08g43334, from *Oryza sativa*; *SiHsfB2b*, Seita.6G236100, from *Setaria italica* and *SbHsfB2b*, Sobic.007G180300, from *Sorghum bicolor*

A full-length cDNA of *ZmHsf11* gene was obtained from maize inbred B73, contained an open reading frame of 1113 bp. This ORF encoded a 370 aa protein with a predicted molecular weight of 39.56 kDa and an isoelectric point of 5.89. Phylogenetic analysis of *Hsf* gene families of *Arabidopsis*, rice, sorghum, millet, and maize revealed that *ZmHsf11* belongs to the HsfB2 subfamily and is most closely related to *ZmHsf03* and *SbHsf21* (*SbHsfB2b*) (Fig. 1A). Multiple sequence alignments indicated that *ZmHsf11* exhibited high similarity with other conserved domains of homologous genes in *Arabidopsis* and other monocots (Fig. 1B). And according to Lin et al., the ZmHsf11 protein contains N-terminal DNA binding domain (DBD), oligomerization domain (OD) and nuclear location signal (NLS), respectively (Fig. 1B) [40]. These results showed that *ZmHsf11* was a member of HsfB2 in maize.

### Expression analysis of *ZmHsf11* in maize

**Fig. 2:**
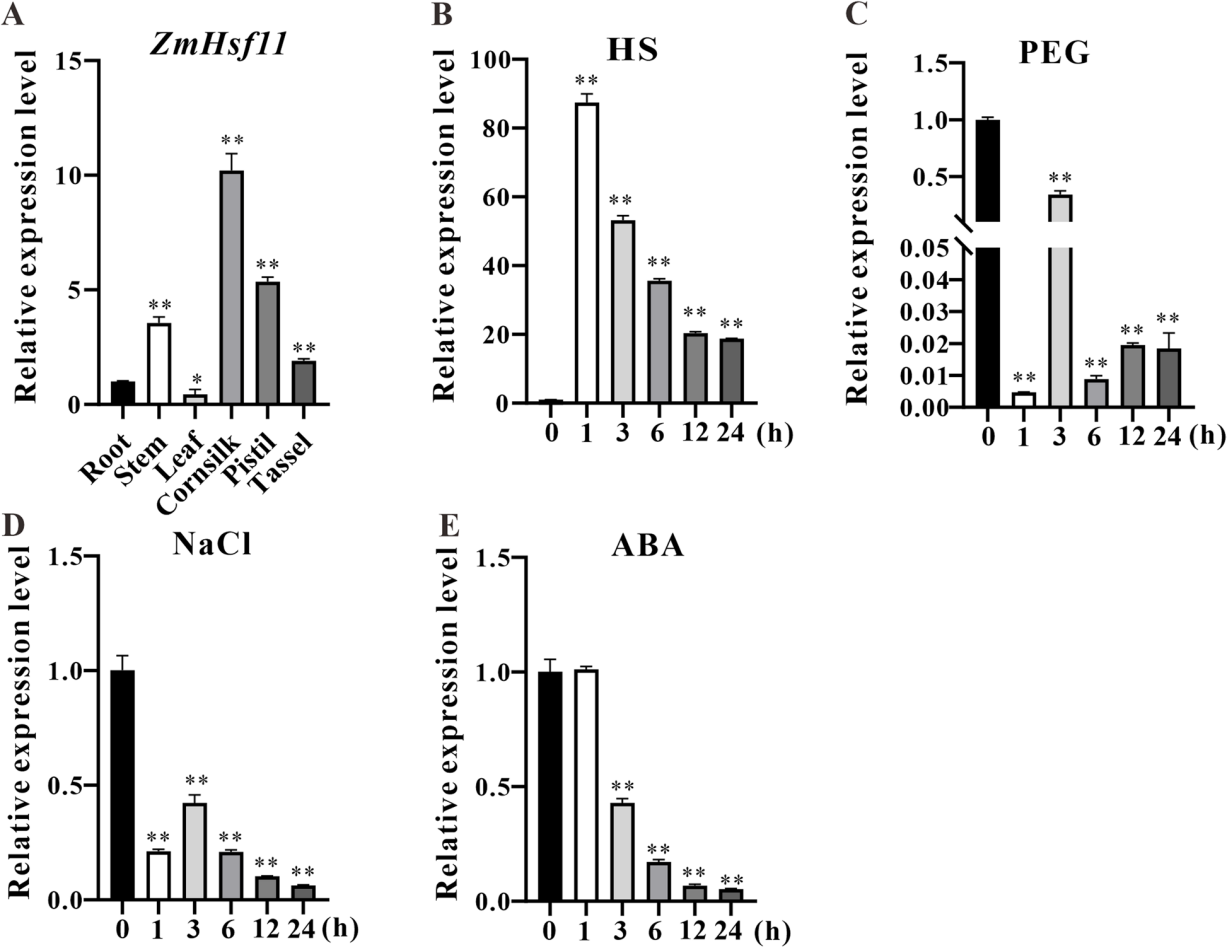
Expression patterns of *ZmHsf11* in maize. **A** Tissue specific expression of *ZmHsf11* in root, stem, leaf, cornsilk, pistil, tassel. **B-E** Expression patterns of *ZmHsf11* during HS, PEG, NaCl and ABA treatments, respectively. The maize *GAPDH* gene was used as the internal reference gene for normalization. Data represent means ± SDs of three independent sample replicates. Significant differences (Student’s t test): *, *P* < 0.05; **, *P* < 0.01

Numerous studies have shown that *Hsf* genes played broad and important roles in stress responses. Therefore, to explore the function of *ZmHsf11* gene, we studied the expression patterns of *ZmHsf11* gene in different tissues and under different stress treatments by RT-qPCR. Different tissues of maize in different growth periods were selected, including root, stem, leave, cornsilk, pistil and tassel. Tissue specific expression analysis showed that *ZmHsf11* gene was expressed in all six tissues of maize, but the expression level in stem, cornsilk and pistil of maize was significantly higher than that in other tissues, and the expression level in leave was significantly lower (Fig. 2A). Subsequently, the expression patterns of *ZmHsf11* under different stresses were investigated. Under heat treatment, the expression of *ZmHsf11* increased first and then decreased (Fig. 2B). The expression of ZmHsf11 was highest at 1 h of heat stress, 85 times higher than that of the untreated group, and then the expression level decreased gradually (Fig. 2B). Under PEG, NaCl and ABA treatments, the expression level of *ZmHsf11* gene was basically lower than that of the untreated group (Fig. 2C, D and E). The above results suggested that *ZmHsf11* potentially involved in abiotic stress pathways such as HS. However, the mechanism of how it participates in the regulation of these pathways need to be further explored.

### Subcellular localization of ZmHsf11

**Fig. 3:**
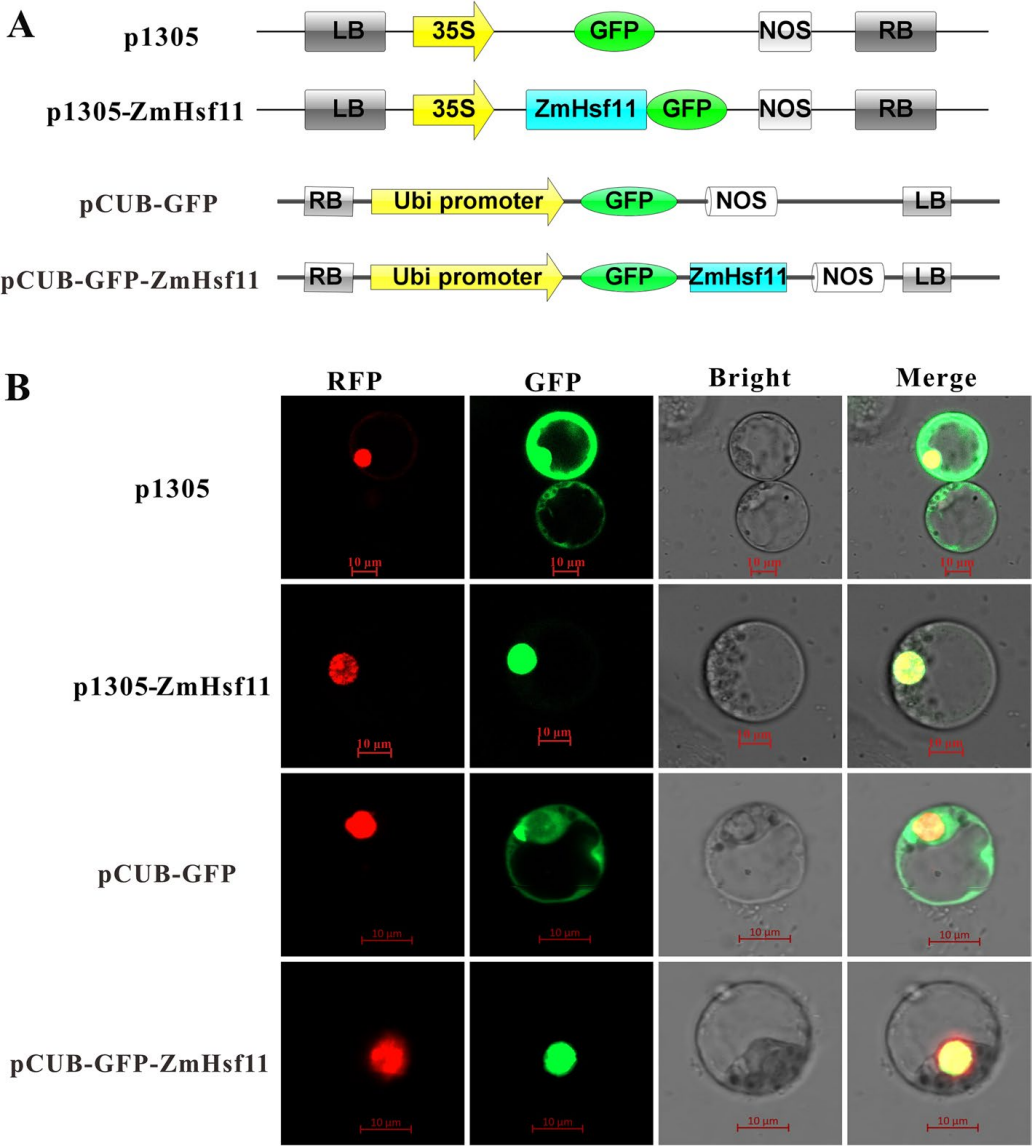
Subcellular localization of ZmHsf11. **A** Schematic representation of constructs used for the subcellular localization of the ZmHsf11 protein. **B** Subcellular localization of the ZmHsf11 protein in maize protoplasts. RFP is a marker for nucleus localization. Bars = 10 µm

It has been reported that heat shock factor (Hsf) contains a nuclear localization signal (NLS), and ZmHsf11 protein was predicted to be a protein located in the nucleus. To investigate the subcellular localization of ZmHsf11, we constructed fusion proteins containing C-terminal and N-terminal green fluorescent protein (GFP) and GFP control proteins co-transfected with the nuclear localization marker pSAT6:mCherry (RFP):VirD2NLS (mCherry), respectively. The GFP signals were observed only in the nucleus containing ZmHsf11-GFP and GFP-ZmHsf11fusion proteins (Fig. 3B). For GFP control proteins, GFP signals were found throughout the whole cell. The results showed that ZmHsf11 protein was localized in the nucleus.

### Transcriptional self-activation activity of *ZmHsf11*

**Fig. 4:**
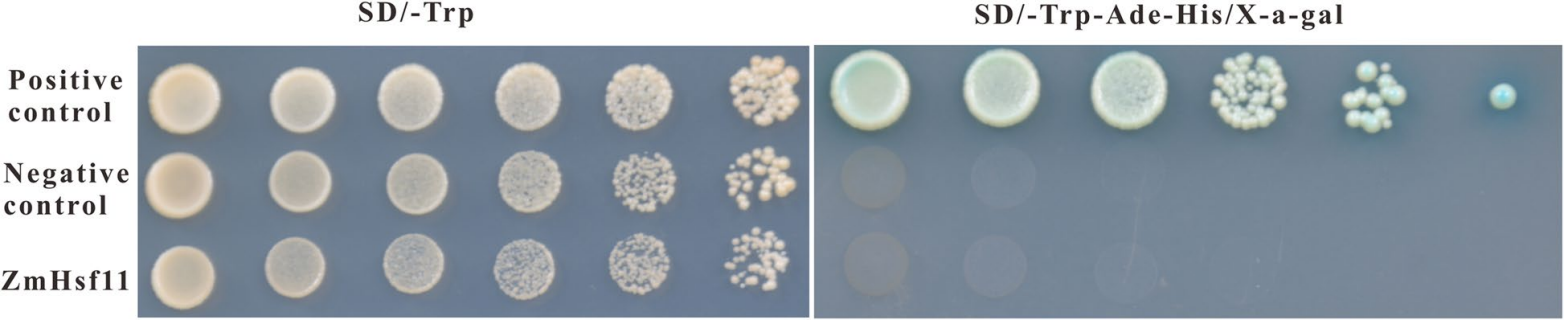
Transcriptional activation activity assay of *ZmHsf11*. Positive control: pGBKT7-53 + pGADT7-T; Negative control: pGBKT7; ZmHsf11: pGBKT7-ZmHsf11

According to previous reports, *ZmHsf11* was a member of the HsfB family without transcriptional self-activation activity. Therefore, the GAL4 system was used to recombine pGBKT7 with *ZmHsf11* gene to determine whether it has transcriptional self-activation activity. The yeast containing *ZmHsf11* grew well on the defect medium SD/Trp − , but could not grow on the defect medium SD/Trp − His − Ade − /X-α-gal, which was consistent with the negative control, and the growth of the positive control plaque was blue on the defect medium SD/Trp − His − Ade − /X-α-gal (Fig. 4). These results indicated that *ZmHsf11* had no transcriptional autoactivation activity in yeast.

### Overexpression of *ZmHsf11* in *Arabidopsis* reduces the tolerance to heat stress

**Fig. 5:**
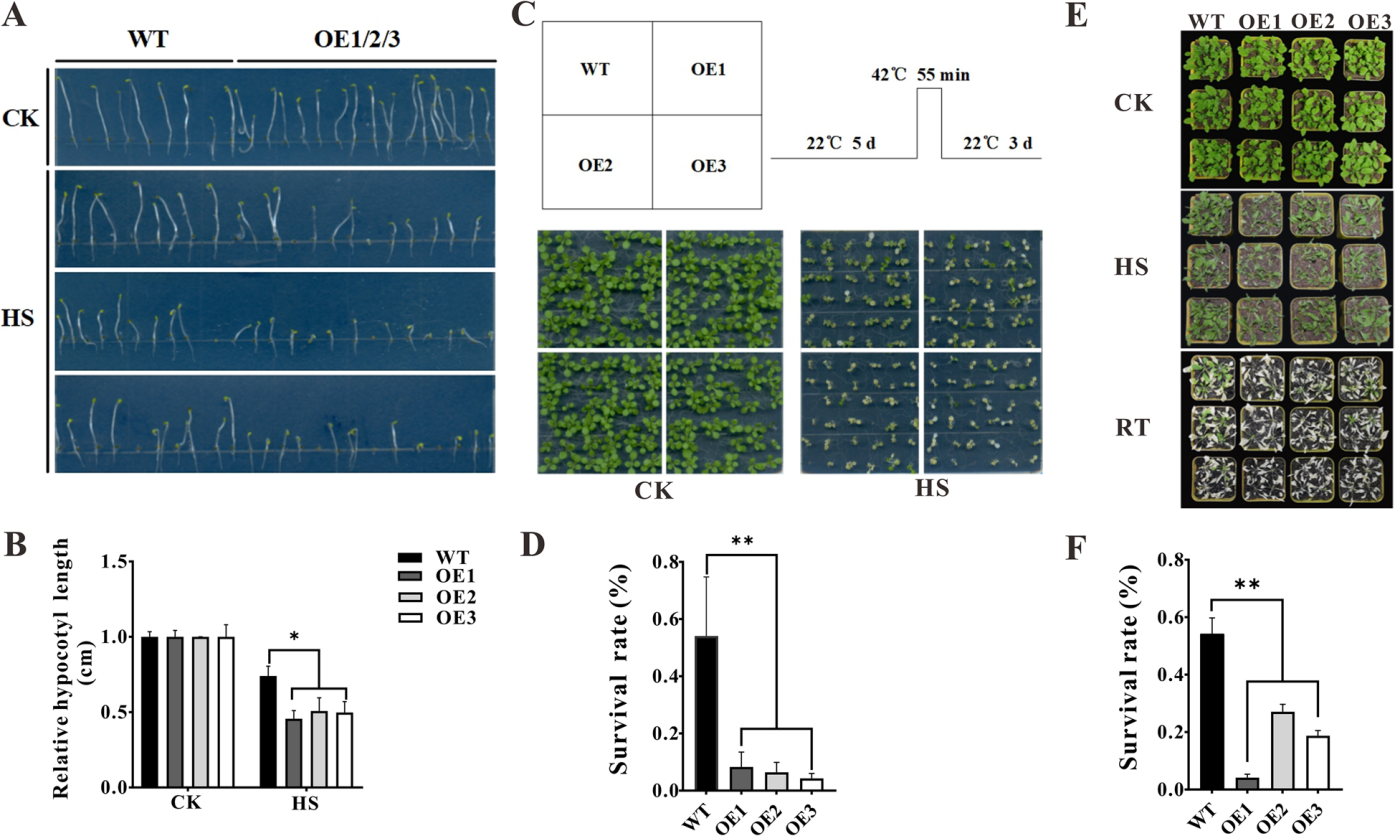
Overexpression of *ZmHsf11* in Arabidopsis reduced the heat stress tolerance. **A** Hypocotyl phenotype under heat stress. **B** Hypocotyl length statistics. **C** Seedling phenotype under heat stress. **D** Seeding survival statistics. **E** Grown seedling phenotype under heat stress. **F** Grown seedling survival statistics. All the values are means (± SDs) from three independent experiments. Significant differences (Student’s t test): *, *P* < 0.05; **, *P* < 0.01

To determine the role of *ZmHsf11* gene in plant response to heat stress, we transformed *ZmHsf11* overexpression vector into *Arabidopsis*, and obtained three overexpressed *Arabidopsis* lines (OE1, OE2, OE3) (Fig. S1). The hypocotyls of overexpressed *ZmHsf11* lines and wild-type plants were treated with heat shock. The hypocotyls of overexpressed *ZmHsf11* lines and wild-type plants were cultured in MS medium at 22 °C for 40 h under dark condition, followed by heat shock at 45 °C for 1 h and dark light recovery at 22 °C for 4 d. After heat treatment, the hypocotyls length of overexpressed *ZmHsf11* lines were significantly lower than that of wild-type plants (Fig. 5A and B). To further study whether the overexpressed plants of *ZmHsf11* in *Arabidopsis* were sensitive to heat, 5 d seedlings and 20 d grown seedlings were heat treated, respectively. The results showed that the survival rate of overexpressed plants was significantly lower than that of the wild-type (Fig. 5C, D, E and F). Therefore, these results suggested that overexpression of *ZmHsf11* in *Arabidopsis* reduced the tolerance of *Arabidopsis* to heat stress.

### Overexpression of *ZmHsf11* in rice reduces the tolerance to heat stress

**Fig. 6:**
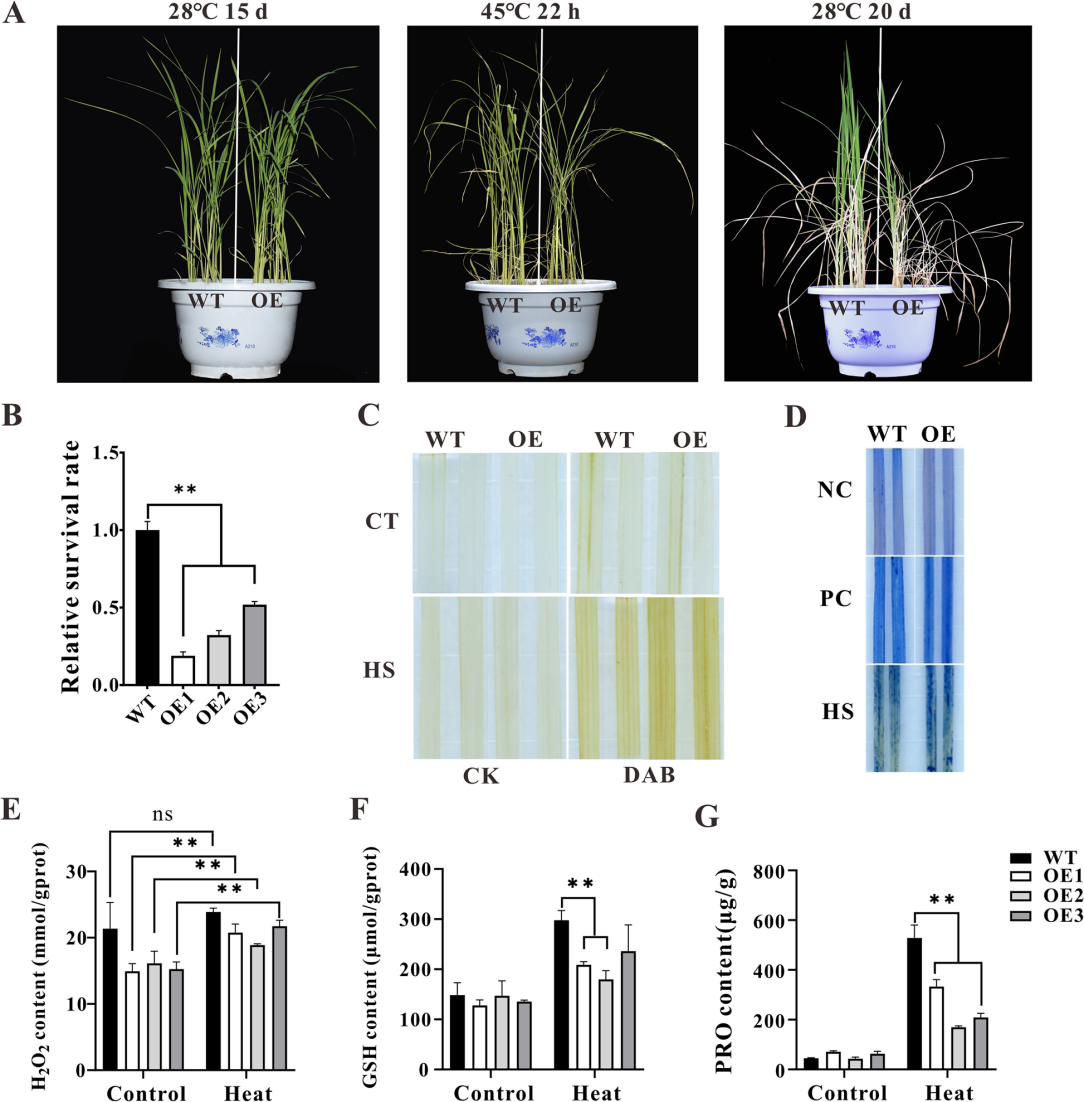
Overexpression of *ZmHsf11* in rice reduced the heat stress tolerance. **A** The phenotypes of wild-type and overexpression of *ZmHsf11* in rice under 45 °C 22 h. **B** Survival statistics. **C** DAB staining of the rice leaves. (CT: 28 °C; HS: 45 °C 22 h; CK: 10 mM NaHPO4). **D** Trypan blue staining of the rice leaves. (NC: room temperature; PC: 95 °C; HS: 45 °C). **E–G** The H_2_O_2_, GSH and proline contents were measured of the rice leaves after heat treatment. Data are means ± SDs of three independent sample replicates. Significant differences (Student’s t test): *, *P* < 0.05; **, *P* < 0.01

To further study the effect of *ZmHsf11* on plant heat sensitivity, we continued to overexpress *ZmHsf11* gene in rice and obtained three transgenic positive rice lines (OE1, OE2, OE3) (Fig. S2). Firstly, seed germination experiments were performed under normal and heat stress condition. After germinating under heat stress treatment for 3 d and recovery at 28 °C for 5 d, plant height was measured to assess the thermotolerance of transgenic and wild-type rice at germination stage. Seeds germinated under 28 °C for 8 d were applied as control. Results showed under control condition, the plant height of only OE1 rice was significantly lower than that of wild-type rice. Whereas, with HS treatment, the plant height of three transgenic plants were significantly lower than that of wild-type plants, indicating that overexpression of *ZmHsf11* gene increased the sensitivity of rice plants to heat stress during germination (Fig. S3). To further investigate the sensitivity of overexpression rice to high temperature, we performed heat treatment for transgenic plants at the seedling stage. Under normal conditions, there was no significant difference between transgenic and wild-type plant seedlings. Overexpression and wild-type rice lines grown at 28 °C for 15 d were heat-treated at 45 °C for 22 h, and after 20 d recovery at 28 °C, showed that the relative survival rate of overexpressed rice lines was significantly lower than that of wild-type plants (Fig. 6A and B). Considering that stress can lead to ROS accumulation in plants, we studied H_2_O_2_ levels in wild-type and transgenic plants under heat treatment by DAB staining, H_2_O_2_ content and GSH content determination. Under normal conditions, there was no significant difference in H_2_O_2_ content between wild-type and transgenic plants. Under heat stress, transgenic plants accumulated significantly more H_2_O_2_ compared with wild-type (Fig. 6C, E and F). Heat stress can lead to cell membrane damage and cell death to some extent. Trypan blue staining experiments showed increased cell death in transgenic plants compared with wild type under heat stress (Fig. 6D). In addition, we also tested the Proline (PRO) content of the wild-type and transgenic plants, and the Proline (PRO) content of the transgenic plants was significantly lower than that of the wild type under heat treatment (Fig. 6G). Under heat stress, photosynthetic attributes such as photosynthetic rate and stomatal conductance decreased. The results found that the net photosynthetic rate and stomatal conductance of the transgenic plants were lower than those of the wild-type after heat treatment, but there was no significant difference. (Fig. S4). The above results were consistent with the previous results of *Arabidopsis*, indicating that overexpression of the *ZmHsf11* gene reduced the tolerance of rice plants to high temperature stress.

### Overexpression of *ZmHsf11* in transgenic plants reduce sensitivity to ABA stress

The plant hormone ABA plays an important role in the response of plants to abiotic stresses such as high temperature. To investigate whether *ZmHsf11* is involved in regulating plant thermal sensitivity through the ABA pathway, we treated seed germination of *ZmHsf11* overexpressed *Arabidopsis* and rice plants with 0.75 µM and 10 µM ABA concentrations, respectively. The results showed that the seed germination rate of *ZmHsf11* gene in *Arabidopsis* overexpressed lines was higher than that of wild-type, and the plant height of overexpressed rice lines was significantly higher than that of wild-type, indicating that *ZmHsf11* gene possibly regulated plant heat stress response by participating in the ABA pathway (Figs. S5 and 7).

**Fig. 7:**
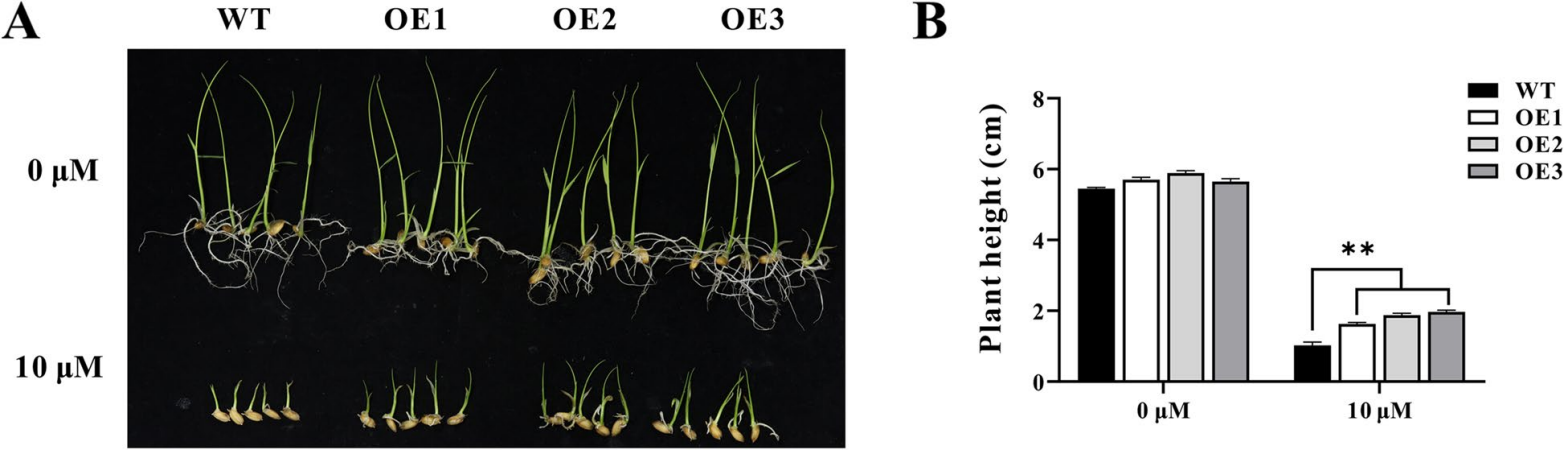
Reduced ABA sensitivity of *ZmHsf11* in rice. **A** Growth performance of wild-type and transgenic rice grown in sterile water containing 10 μM ABA concentrations after 10 d. **B** Measurements of plant heights of wild-type and *ZmHsf11* transgenic seedlings. Data are means ± SDs (n = 10). Significant differences (Student’s t test): **, *P* < 0.01

### Expression analysis of stress-related genes of *ZmHsf11* in transgenic rice

**Fig. 8:**
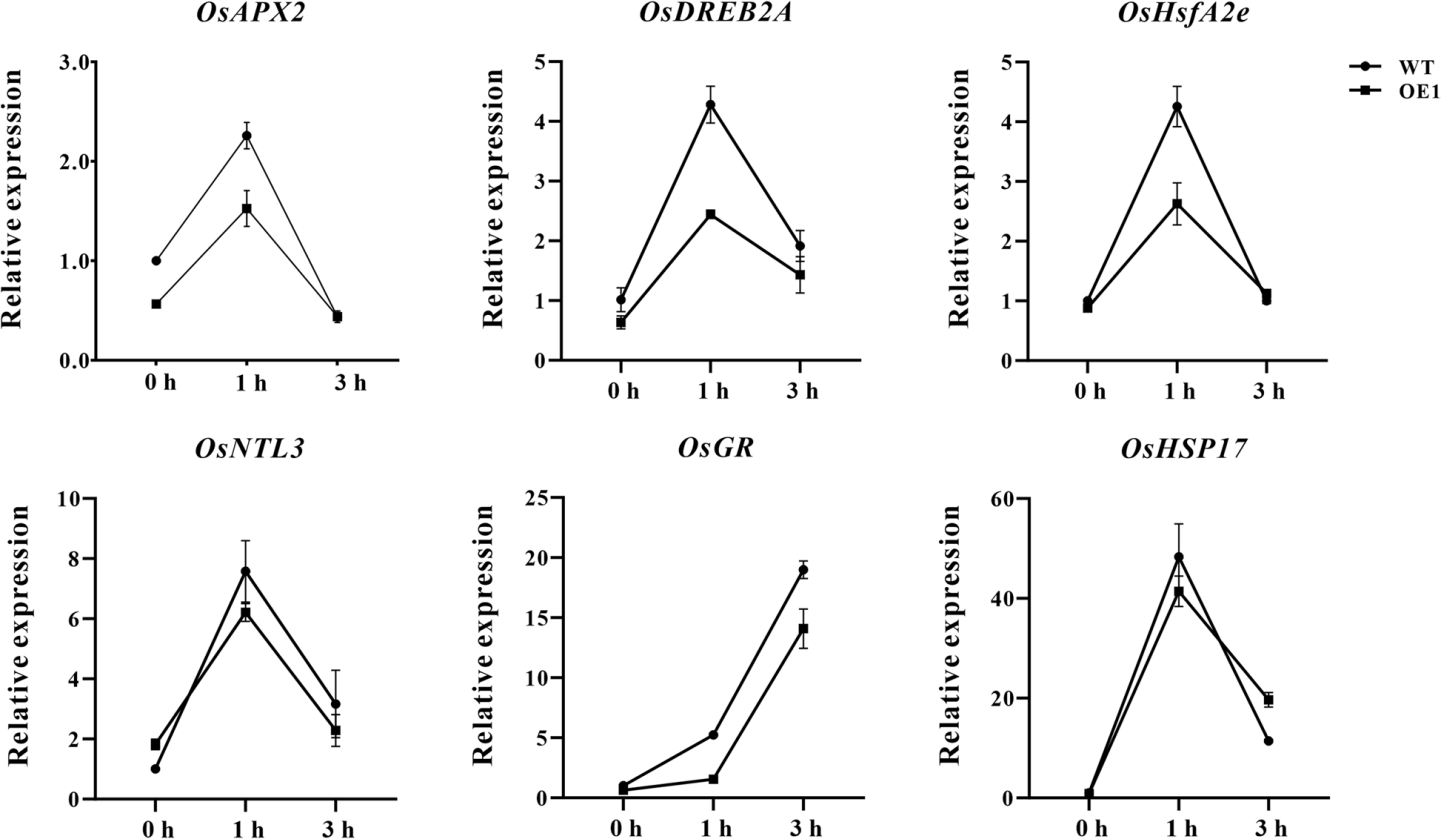
Expression patterns of oxidative-stress-related genes in wild-type and overexpression line 1(OE1) in rice under normal condition and heat treatment. The expression levels of these genes were analyzed by RT-qPCR. Data are means ± SDs of three independent sample replicates

To further explore the functional molecular mechanism of *ZmHsf11* under heat stress, we detected the expression of oxidative stress related genes *DREB2A, HsfA2e, NTL3, HSP17, HSP18, GR, APX2* and *Rab7* under heat stress by RT-qPCR assays. Under normal growth conditions, there was no significant difference in the expression of these genes between *ZmHsf11* overexpressed and wild-type rice. However, under heat treatment, the expression levels of *APX2, DREB2A, HsfA2e, NTL3, GR* and *HSP17* in transgenic rice were significantly lower than those in wild-typerice (Fig. 8). The transcription levels of the other 2 genes (*HSP18* and *Rab7*) were not significantly changed (data not shown). These results indicated that *ZmHsf11* negatively regulated oxidative stress-related genes *APX2, DREB2A, HsfA2e, NTL3, GR* and *HSP17* under heat stress.

## Discussion

Plant responds to varied environmental stresses through complex signaling networks, and heat shock transcription factors (Hsfs) play a crucial role in plant response to abiotic stresses. Class A Hsfs contained AHA activation domain at the C-terminus and have been reported as positive regulators of stress tolerance [10, 41]. However, class B Hsfs contained a BRD inhibitory domain at the C-terminus and no AHA activation domain, and were identified as negative regulators of stress tolerance [42]. For example, *HsfB1* and *HsfB2b* had inhibitory activity and negatively regulated stress tolerance in *Arabidopsis* and soybean [32, 43]. In maize, current studies have reported that there were few *Hsf* gene members, such as class A members *ZmHsf01, ZmHsf05* and *ZmHsf06* that were tolerance under abiotic stress, and a class B member *ZmHsf08* that was sensitivity to salt and drought [39, 44–46]. In this study, we reported the characterization of *ZmHsf11*, a member of class B Hsfs in maize. Our data suggested that *ZmHsf11* was probably a negative regulator involved in high temperature stress tolerance.

Phylogenetic tree analysis showed that *ZmHsf11* shared homology with *HsfB2* members of other species (Fig. 1A), revealing that *ZmHsf11* belonged to the *HsfB2* subfamily. Multiple alignment of the amino acid sequences of ZmHsf11 and other homologous proteins found that ZmHsf11 contained a conserved DNA binding domain (DBD), an intermediate oligomerization domain (OD) and a nuclear localization signal (NLS), lacked nuclear export signal (NES) and activation domain (AHA) (Fig. 1B). This may lead to changes in its structure and function, thus acting as a negative regulator in the opposite function of class A members. The structure was further verified by yeast transcriptional autoactivation assay and protoplast transient expression subcellular localization experiment. The absence of its AHA domain made the gene lack transcriptional autoactivation activity, and the presence of NLS allowed the gene to localize in the nucleus (Figs. 3 and 4). The above results revealed that ZmHsf11 was a highly conserved Hsf protein that possibly needed to interact with other proteins to activate its transcriptional ability.

Studies have shown that *Hsfs* are involved in a variety of abiotic stress pathways and ABA-mediated stress pathways. Overexpression of *FaHsfA2c* in tall fescue enhanced the tolerance of plants to heat stress through inducing high expression of heat stress-related genes in vivo and enhancing photosynthesis ability [47]. And overexpression of *ZmHsf04* enhanced tolerance to heat and salt stress in transgenic Arabidopsis [48]. The *HsfA6b* in *Arabidopsis* enhanced plant tolerance to heat, salt and drought stress through an ABA-mediated pathway [49]. The *MdHSFA8a* promoted the accumulation of flavonoids and removed reactive oxygen species, thus improving the drought tolerance of plants [50]. *Arabidopsis HsfB1* and *HsfB2b* regulated the expression of the defensin gene *Pdf1.2 a/b* in plants and significantly enhanced the disease resistance of *hsfb1-hsfb2b* double mutants [31]. In this study, we observed the expression levels of *ZmHsf11* under different abiotic stresses and ABA hormone treatment, and the expression patterns of *ZmHsf11* in different tissues were surveyed. Like other members of *Hsfs*, *ZmHsf11* was also strongly induced by heat stress, but the expression level gradually decreased with increasing heat treatment time (Fig. 2B). However, the expression of *ZmHsf11* was inhibited under NaCl, PEG and ABA treatments (Fig. 2C, D and E). Therefore, the results showed that *ZmHsf11* potentially involved in different biological processes as a negative regulator of *Hsfs* in plants. The expression level of *ZmHsf11* gene in maize was identified by tissue expression pattern, and it was found that *ZmHsf11* gene was highly expressed in stem, cornsilk and pistil (Fig. 2A), which may indicate that *ZmHsf11* gene was involved in plant growth and development and regulated reproductive organs tissue response to heat stress.

To determine the function of *ZmHsf11* gene in plant responses to heat stress, *ZmHsf11* gene was overexpressed in *Arabidopsis* and rice. Compared with wild-type, overexpression of *ZmHsf11* gene reduced the tolerance of transgenic plants to heat stress (Figs. 5 and 6A, B and S3). This may be related to the accumulation of ROS in plants after heat stress [51]. Reactive oxygen species (ROS) play a key role in plant adaptation to abiotic stress [52]. ROS increases in cells, causing oxidative damage to cell membranes, proteins, RNA and DNA molecules, and even oxidative damage to cells [53]. Therefore, regulation of ROS under high temperature stress is crucial. Proline acts as an important antioxidant, reducing oxidative damage under stressful conditions [54]. In this study, the transgenic rice accumulated more H_2_O_2_ after heat treatment (Fig. 6C, E and F), while the proline content was significantly lower than that of the wild-type (Fig. 6G). Meanwhile, heat stress can cause cell membrane damage and cell death to a certain extent. Trypan blue staining showed that the cell death of transgenic plants increased significantly under heat stress (Fig. 6D). Therefore, these results explained that *ZmHsf11* may play a negative regulatory role in plant response to heat stress. ABA signaling pathway has been reported to be related to abiotic stresses such as drought, salt, and high temperature [55, 56]. In this study, the inducible expression pattern analysis showed that *ZmHsf11* had a similar expression trend to heat shock and ABA (Fig. 2B and E), and the *ZmHsf11* transgenic plants were also less sensitive to ABA during seed germination (Fig. 7 and Fig. S4). These results probably revealed the tandem of abiotic stresses such as high temperature and ABA, thereby regulating the homeostasis in plants under stress conditions. This speculation remains to be explored by further experiments.

To elucidate the potential molecular mechanism of the reduced heat tolerance in plants overexpressing *ZmHsf11*, we investigated the expression of some oxidative stress-related genes *DREB2A, HsfA2e, NTL3, HSP17, HSP18, GR, APX2* and *Rab7* in rice. *DREB* gene played a positive role in the expression of drought, high salt and low temperature responsive genes [57]. *OsHsfA2e* enhanced heat and salt tolerance in transgenic plants [58]. *OsNTL3* regulated the expression of genes involved in ER protein folding and other processes, thereby regulating plant heat tolerance [59]. Overexpression of *OsHsp17* and *OsHsp18* enhanced the resistance of rice to abiotic stress [60, 61]. *OsGR* increased the thermotolerance of yeast strains [62]. *APX2* was a pleiotropic protein involved in H_2_O_2_ homeostasis, chloroplast protection, maintenance of plant configuration and fertility [63]. Overexpression of *OsRab7* gene improved the drought and heat tolerance of rice, and thus increased rice yield [64]. Under heat stress, the expression levels of *APX2, DREB2A, HsfA2e, NTL3, GR* and *HSP17* were decreased in transgenic rice, suggesting that *ZmHsf11* may negatively regulate oxidative stress-related genes (Fig. 8). These results indicated that *ZmHsf11* negatively regulated the heat stress response by inhibiting the expression levels of oxidative stress-related genes.

## Conclusions

A maize HsfB2 member, *ZmHsf11*, was cloned and characterized.. Overexpression of *ZmHsf11* gene in *Arabidopsis* and rice reduced plant tolerance to heat stress through negatively regulating the expression of oxidative stress-related genes, and this was also associated with the accumulation of ROS levels and the reduction of proline content.

## Materials and methods

### Plant materials and stress treatments

Maize B73 inbred lines were germinated and grown for 14 d in a greenhouse with a 16/8 h light/dark at 28 ℃/24 ℃, then treated with 42 ℃, 200 mM NaCl, 20% PEG6000 and 100 µM ABA. Samples were taken at 0 h, 1 h, 3 h, 6 h, 12 h and 24 h for each treatment. Meanwhile, root, stem and leaf samples were taken, and cornsilk, pistil and tassel were taken respectively when they grew to the flowering stage, three plants of each sample are mixed for sampling and quick-frozen with liquid nitrogen, stored at -80 ℃ for RNA extraction.

Zhonghua11 used for transgenic rice was consistent with the maize growth greenhouse. The Columbia type used for transgenic *Arabidopsis* was grown at 22 ℃/20 ℃ with a 16/8 h light/dark. *Arabidopsis* seeds were grown and identified on MS medium, and seedlings were then transferred to a mixture of peat and vermiculite at 14 d.

### Cloning and sequence analysis of *ZmHsf11*

In the plant genome database website (https://phytozome.jgi.doe.gov/pz/portal.html), the full-length CDS sequence and amino acid sequence of the maize *ZmHsf11* gene were obtained. The amino acid sequences of the *ZmHsf11* homologous genes were obtained from the NCBI website (https://www.ncbi.nlm.nih.gov/). The amino acid sequence of *ZmHsf11* was analyzed by ExPASy website (https://web.expasy.org/protparam/), and the sequences of *ZmHsf11* and its homologous genes were compared by DNAMAN software [65]. Amino acid sequences of the *Hsf* gene families in *Arabidopsis*, rice, sorghum, millet and maize were obtained using the Phytozome (https://phytozome-next.jgi.doe.gov/pz/portal.html) and Pfam websites (http://pfam.xfam.org/). The phylogenetic tree was constructed by NJ method using MEGA X software, and bootstrap analysis was performed, with a total of 1000 replicates [66]. According to the obtained full-length cDNA sequence of *ZmHsf11*, specific primers for the *ZmHsf11* gene were designed using Primer5 software, and the full-length *ZmHsf11* gene was amplified by PCR assay. The PCR product was purified and cloned into the pEASY-Blunt simple vector for sequencing. Primers for ZmHsf11-F and ZmHsf11-R were shown in Supplementary Table S1.

### Quantitative real‑time PCR and semi real‑time PCR analysis

Total RNA was extracted by Trizol method using RNA isolater Total RNA Extraction Reagent (Vazyme R401-01), and cDNA for RT-qPCR was synthesized using HiScript III RT SuperMix for qPCR (+ gDNA wiper) (Vazyme R323-01), cDNA for RT-PCR was synthesized using HiScript III 1st Strand cDNA Synthesis Kit (+ gDNA wiper) (Vazyme R312-01). RT-qPCR was performed using AceQ qPCR SYBR Green Master Mix (Vazyme Q111-02) on the LightCycler480 instrument (Roche). The GAPDH and Actin genes were used as internal control for maize. The relative expression level was calculated using the 2^−ΔΔCT^ method [67]. All the primers used for RT-qPCR were listed in Supplementary Table S1.

### Subcellular localization of the ZmHsf11 protein

The coding sequence of *ZmHsf11* gene without terminator was linked into p1305-GFP vector by double digestion method (Xba I/Sma I). And the coding sequence of *ZmHsf11* gene was ligated into pCUB-GFP vector by homologous recombination method, and primers are shown in Supplementary Table S1. The p1305-ZmHsf11-GFP, pCUB-GFP- ZmHsf11 recombinant plasmids and p1305-GFP and pCUB-GFP empty vectors were co-transformed into protoplasts together with the nuclear localization signal plasmid pSAT6:mCherry (RFP):VirD2NLS by transient transformation technology. After culturing in the dark at 28 °C for 18 h, the protoplasts were observed by laser scanning microscopy (LSM800, Carl Zeiss, Jena, Germany).

### Transcriptional self-activation activity assay in yeast

The coding sequence of the *ZmHsf11* gene was homologous recombined into the pGBKT7 vector using EcoR I restriction enzyme and ClonExpress II One Step Cloning Kit (Vazyme C112-01), and primers are shown in Supplementary Table S1. The recombinant plasmid pGBKT7-ZmHsf11 was transformed into yeast strain Y2H Gold by PEG/LiAc method. Positive control was pGBKT7-53 and pGADT7-T, and negative control was pGBKT7 vector. The transformed yeast cells were serially diluted on SD/Trp − and SD/Trp − Leu − Ade − /X-α-Gal plates, respectively, and the yeast cells were grown at 30 °C in the dark for 3 d.

### Generation of *ZmHsf11* transgenic plants

To construct the overexpression vector, the coding sequence of *ZmHsf11* gene was linked to the p1301a vector (modified in this experiment) by double digestion method (Kpn I/Pst I) to obtain the recombinant plasmid p1301a-*ZmHsf11*. The recombinant plasmid was transferred into *Arabidopsis* and rice by agrobacterium-mediated method to obtain transgenic T_0_ generation plants. Transgenic T_1_ generation plants were screened by hygromycin, GUS staining and semi real‑time PCR assays to obtain multiple positive lines. Three of the overexpressing *Arabidopsis* and rice lines (OE1, OE2, OE3) were selected and cultured to obtain T_3_ homozygous overexpression plants for subsequent experiments.

### Heat stress tolerance assays

The seeds of T_3_ generation of three overexpressed strains and wild-type of *Arabidopsis* were placed on MS plates and sealed. Each plate was placed vertically, wrapped with tin foil, and grown in the dark at 22 °C/20 °C for 2 d. Then, heat treatment at 45 °C for 1 h in an artificial climate box in the dark, then wrapped it with tin foil, and placed in greenhouse for dark growth for 3–4 d. The hypocotyl length of different strains was observed and statistical data were obtained.

The seeds of T_3_ generation of three overexpressed strains and wild-type of *Arabidopsis* were placed on MS plates and sealed. Each plate was placed horizontally with 36 seeds at each plant point. The seeds were grown at 22 °C /20 °C for 5 d in a 16/8 h, light/dark greenhouse, used a water bath at 42 °C for 55 min, cooled to room temperature, and then put it back in the greenhouse for about 3 d to observe the survival rate of seedlings of different lines and make statistics.

Three *Arabidopsis* overexpression lines T_3_ generation seeds and wild-type were grown in MS plate at 22 °C/20 °C, 16/8 h, light/dark greenhouse for 10 d, and then transplanted to pots with vermiculite: black soil = 2: 1, continued to grow for 10 d, used artificial climate chamber, under 45 °C light conditions, 6.5 h treatment, replaced in the greenhouse for 4–5 d to recover cultivation, observed the survival rate of seedlings of different lines, and statistical data.

The seeds of the T_3_ generation of the three overexpressed rice lines and the wild-type seeds of Zhonghua 11 were fully swollen and placed in a moderate heat treatment at 35 °C for 3 d and a recovery at 28 °C for 5 d. The plant height of rice seeds of different lines was measured by a ruler.

The seeds of the T_3_ generation of the three overexpression lines of rice and the wild-type seeds of Zhonghua11 were transplanted into small round pots after 8 d of germination, which were placed in a 28 °C/24 °C, 16/8 h, light/dark greenhouse for 15 d. In an artificial climate box, under the light condition of 45 °C, treated for 22 h, and then placed in the greenhouse to resume cultivation for 20 d. The survival rate of rice seedlings of different lines was observed and the statistics were collected.

### Physiological analysis of plants in response to heat stress

The 15-day-old rice seedlings were heat-treated at 45 °C for 22 h, and the physiological features of overexpression and wild-type rice were determined respectively. The same parts of untreated and heat-treated rice leaves were taken for DAB and trypan blue staining, as described by Zhang et al. [68]. And the contents of hydrogen peroxide (H_2_O_2_), reduced glutathione (GSH) and proline (PRO) were determined according to the protocol of Nanjing Jiancheng Bioengineering Institute. Net photosynthetic rate and stomatal conductance were measured using a photosynthesis instrument CIRAS-3.

### ABA sensitivity analysis

The vernalized *Arabidopsis* seeds were sown on MS medium containing 0.75 μM ABA, with 36 seeds in each plant line, and placed in the *Arabidopsis* greenhouse, grown for 8 d. The germination status of seeds was observed, and the germination rate was calculated. Sterilized rice seeds were cultured in sterile water at 30 °C for 2 d in the dark. Then, rice seeds of the same size were selected and continued to be cultured in sterile water containing 10 μM ABA in a greenhouse at 28 °C for 8 d. The heights of different lines were measured and the data were counted.

### Analysis of stress-related genes regulated by *ZmHsf11*

The expression levels of oxidative stress-related genes *DREB2A, HsfA2e, NTL3, HSP17, HSP18, GR, APX2* and *Rab7* in overexpressed and wild-type rice lines were analyzed by RT-qPCR experiments. For the overexpression line OE1 and wild-type rice, samples were taken at 45 °C for 0 h, 1 h and 3 h, respectively. The other steps were the same as above.

## Supplementary Information

**Additional file 1:** **Figure 1.** Original image of semi-quantitative RT-PCR analysis of *ZmHsf11* expression levels in WT, p1301a vector and three T_1_ transgenic lines in Arabidopsis. **Figure 2.** Original image of semi-quantitative RT-PCR analysis of *ZmHsf11* expression levels in WT and three T_1_ generation transgenic lines in rice.

**Additional file 2:** **Figure S1.** Identification of *ZmHsf11* overexpression positive transgenic Arabidopsis. (A) Schematic representation of a ZmHsf11-containing vector for overexpression transformation. (B) GUS staining of different tissues of positive lines, including seeding, leaf and stem, inflorescence, and silique. (C) Semi-quantitative RT-PCR analysis of *ZmHsf11* expression levels in wild-type, p1301a vector and three T_1_ generation transgenic lines in Arabidopsis. *AtActin* (*AtUBQ5*) was used as an internal control. **Figure S2.** Identification of *ZmHsf11* overexpression positive transgenic rice. (A) GUS staining of different tissues of positive lines, including root, stem and leaves. (B) Semiquantitative RT-PCR analysis of *ZmHsf11* expression levels in wild-type and three T_1_ generation transgenic lines in rice. *OsActin* (*OsUBQ5*) was used as an internal control. **Figure S3.** Enhanced heat sensitivity during germination in *ZmHsf11* transgenic rice. (A) Seed germination status of wild-type and transgenic plants after treatment at 35 °C for 3 d and recovery at 28 °C for 5 d. (B) Plant height measurements during germination period of wild-type and *ZmHsf11* transgenic seeds. Data are mean ± standard deviation (*n* = 15). **, *P* < 0.01. **Figure S3.** Enhanced heat sensitivity during germination in *ZmHsf11* transgenic rice. (A) Seed germination status of wild-type and transgenic plants after treatment at 35 °C for 3 d and recovery at 28 °C for 5 d. (B) Plant height measurements during germination period of wild-type and *ZmHsf11* transgenic seeds. Data are mean ± standard deviation (*n* = 15). **, *P* < 0.01. **Figure S4.** Physiological indicators of *ZmHsf11* transgenic and wild-type rice under heat stress. (A) Net photosynthetic rate. (B) Stomatal conductance. Data are means ± SDs of three independent sample replicates. *, *P* < 0.05. **Figure S5.** Reduced ABA sensitivity of ZmHsf11 in Arabidopsis. Growth performance of wild-type and transgenic plants grown on MS medium containing 0.75 μM ABA concentrations after 8 d. Measurements of germination rate of wild-type and *ZmHsf11* transgenic seedlings. Data are means ± SDs (*n* = 36). *, *P* < 0.05; **, *P* < 0.01. **Table S1.** Primer sequences used in this study.

## Data Availability

All data generated or analysed during this study are included in this published article and its supplementary information files. However, the sequence data in this study can also be accessed at https://www.ncbi.nlm.nih.gov/gene/?term=GRMZM2G098696. In addition, all databases used in this study are open for public and the links are as follows: Phytozome: https://data.jgi.doe.gov/refine-download/phytozome?q=Arabidopsis+thaliana ExPASy: https://web.expasy.org/cgi-bin/protparam/protparam1?A0A804NZV2@noft@.

## Abbreviations

Hsf: Heat shock transcription factor
ABA: Abscisic acid
ROS: Reactive oxygen species
H_2_O_2_: Hydrogen peroxide
GSH: Glutathione
