# Supplementary material for: A maize heat shock factor ZmHsf11 negatively regulates heat stress tolerance in transgenic plants
Source: BMC Plant Biol. 2022 Aug 20;22:406. doi: 10.1186/s12870-022-03789-1 (PMC9392289; doi:10.1186/s12870-022-03789-1)

**Figure 1.** Original image of semi-quantitative RT-PCR analysis of *ZmHsf11* expression levels in WT, p1301a vector and three $T_1$ transgenic lines in *Arabidopsis*.

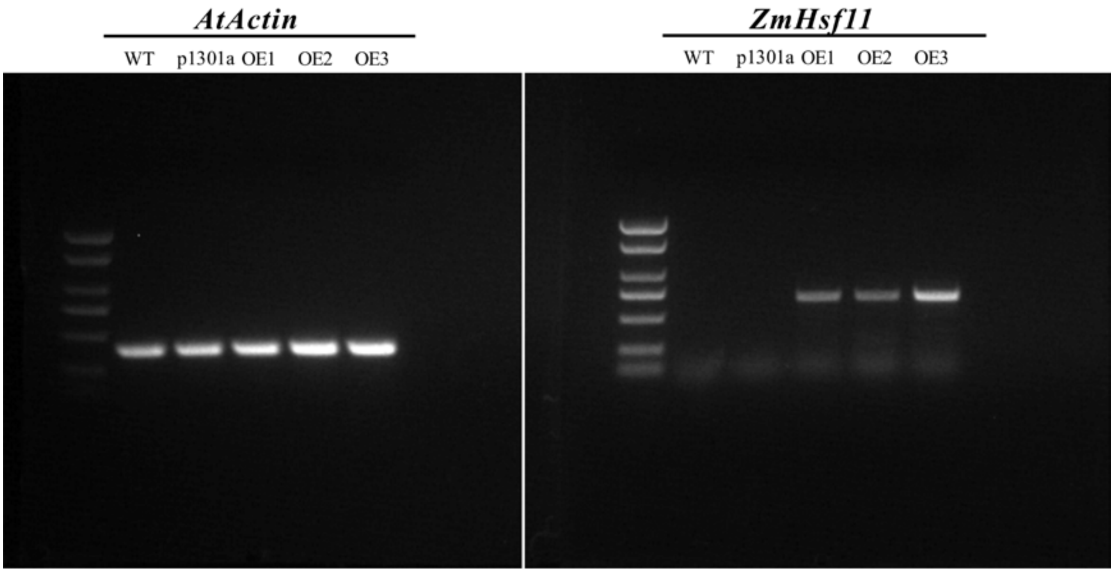


**Figure 2.** Original image of semi-quantitative RT-PCR analysis of *ZmHsf11* expression levels in WT and three $T_1$ generation transgenic lines in rice.

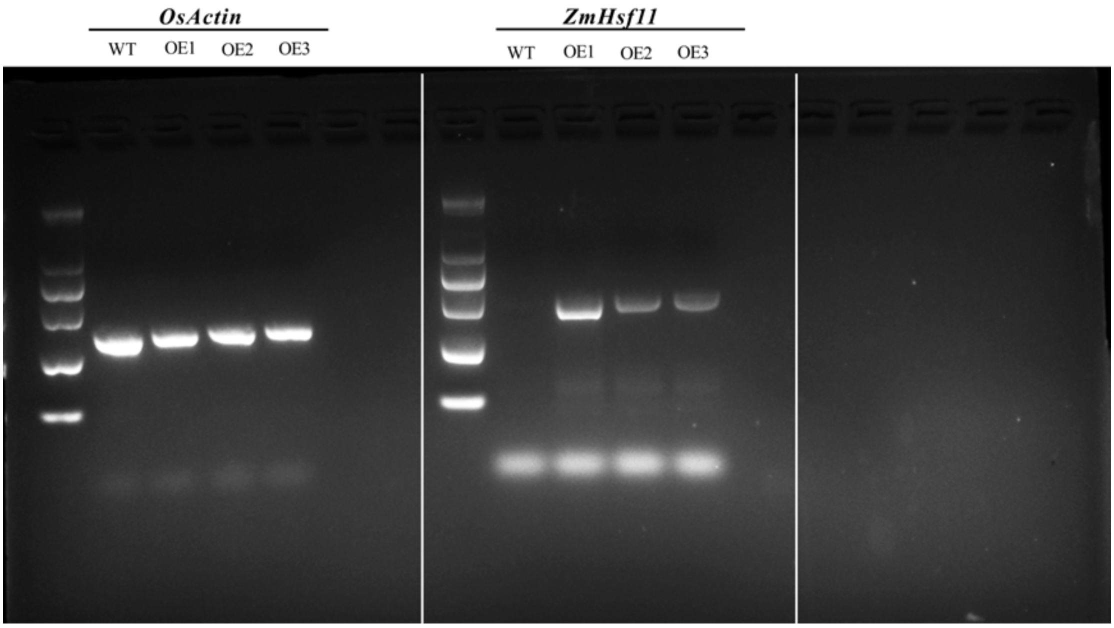

Supplement: Supplementary file 1 — Additional file 1: Figure 1. Original image of semi-quantitative RT-PCR analysis of ZmHsf11 expression levels in WT, p1301a vector and three T1 transgenic lines in Arabidopsis. Figure 2. Original image of semi-quantitative RT-PCR analysis of ZmHsf11 expression levels in WT and three T1 generation transgenic lines in rice. [file 12870_2022_3789_MOESM1_ESM.pdf]
