# Supplementary material for: A maize heat shock factor ZmHsf11 negatively regulates heat stress tolerance in transgenic plants
Source: BMC Plant Biol. 2022 Aug 20;22:406. doi: 10.1186/s12870-022-03789-1 (PMC9392289; doi:10.1186/s12870-022-03789-1)
Supplement: Supplementary file 2 — Additional file 2: Figure S1. Identification of ZmHsf11 overexpression positive transgenic Arabidopsis. (A) Schematic representation of a ZmHsf11-containing vector for overexpression transformation. (B) GUS staining of different tissues of positive lines, including seeding, leaf and stem, inflorescence, and silique. (C) Semi-quantitative RT-PCR analysis of ZmHsf11 expression levels in wild-type, p1301a vector and three T1 generation transgenic lines in Arabidopsis. AtActin (AtUBQ5) was used as an internal control. Figure S2. Identification of ZmHsf11 overexpression positive transgenic rice. (A) GUS staining of different tissues of positive lines, including root, stem and leaves. (B) Semiquantitative RT-PCR analysis of ZmHsf11 expression levels in wild-type and three T1 generation transgenic lines in rice. OsActin (OsUBQ5) was used as an internal control. Figure S3. Enhanced heat sensitivity during germination in ZmHsf11 transgenic rice. (A) Seed germination status of wild-type and transgenic plants after treatment at 35 °C for 3 d and recovery at 28 °C for 5 d. (B) Plant height measurements during germination period of wild-type and ZmHsf11 transgenic seeds. Data are mean ± standard deviation (n = 15). **, P < 0.01. Figure S3. Enhanced heat sensitivity during germination in ZmHsf11 transgenic rice. (A) Seed germination status of wild-type and transgenic plants after treatment at 35 °C for 3 d and recovery at 28 °C for 5 d. (B) Plant height measurements during germination period of wild-type and ZmHsf11 transgenic seeds. Data are mean ± standard deviation (n = 15). **, P < 0.01. Figure S4. Physiological indicators of ZmHsf11 transgenic and wild-type rice under heat stress. (A) Net photosynthetic rate. (B) Stomatal conductance. Data are means ± SDs of three independent sample replicates. *, P < 0.05. Figure S5. Reduced ABA sensitivity of ZmHsf11 in Arabidopsis. Growth performance of wild-type and transgenic plants grown on MS medium containing 0.75 μM ABA concentratio [file 12870_2022_3789_MOESM2_ESM.pdf]

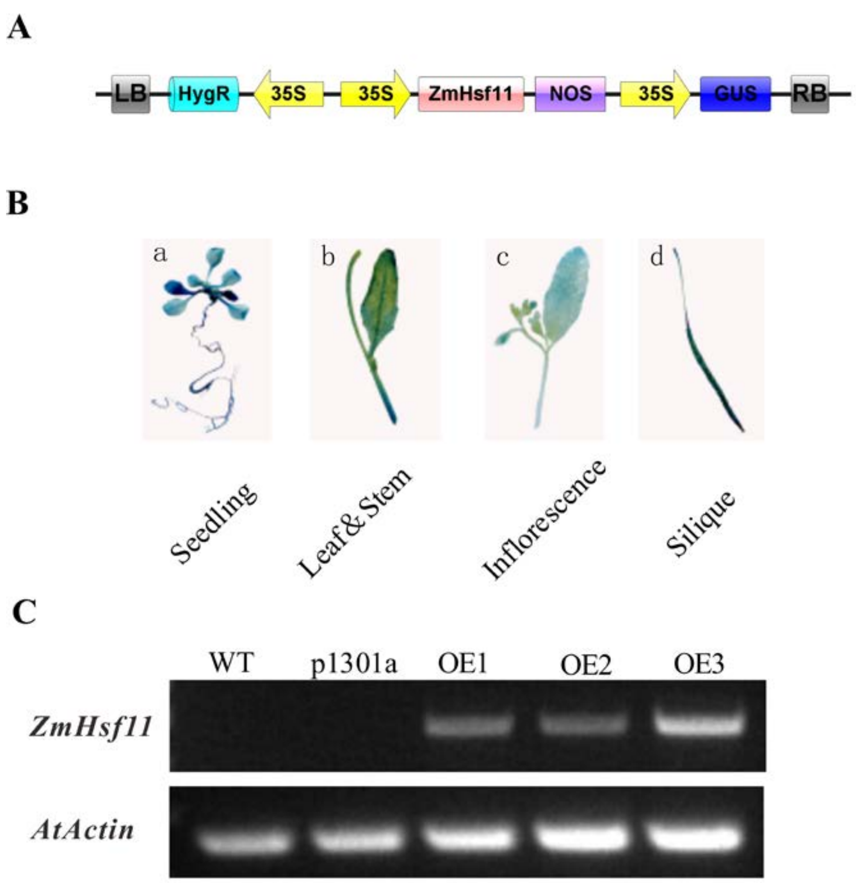

Figure S1. Identification of *ZmHsf11* overexpression positive transgenic *Arabidopsis*. (A) Schematic representation of a ZmHsf11-containing vector for overexpression transformation. (B) GUS staining of different tissues of positive lines, including seeding, leaf and stem, inflorescence, and silique. (C) Semi-quantitative RT-PCR analysis of *ZmHsf11* expression levels in wild-type, p1301a vector and three T_1 generation transgenic lines in *Arabidopsis*. *AtActin* (*AtUBQ5*) was used as an internal control.

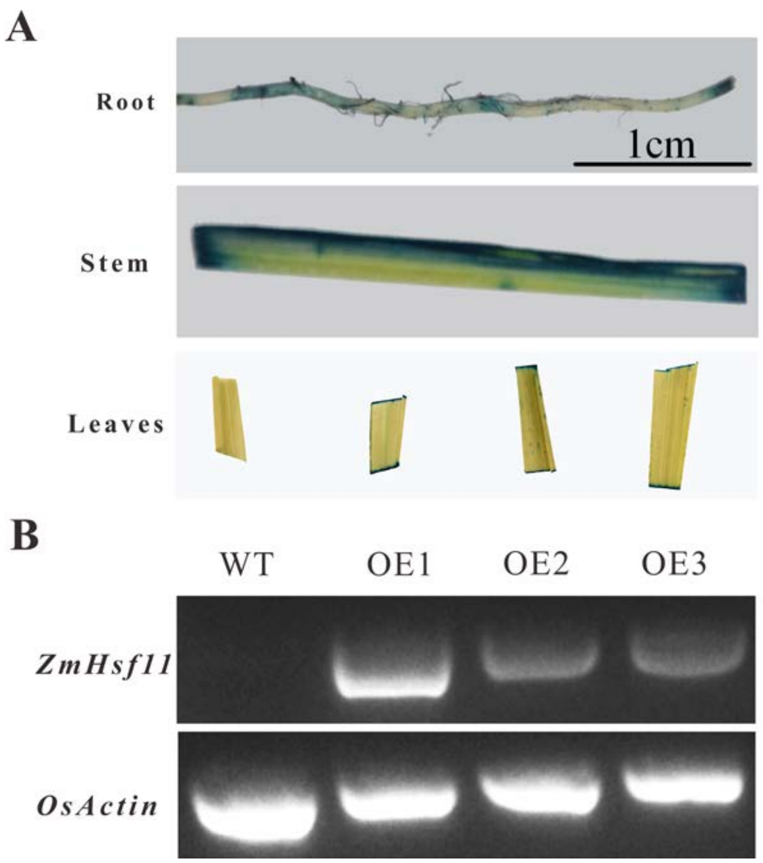

Figure S2. Identification of *ZmHsf11* overexpression positive transgenic rice. (A) GUS staining of different tissues of positive lines, including root, stem and leaves. (B) Semi-quantitative RT-PCR analysis of *ZmHsf11* expression levels in wild-type and three T_1 generation transgenic lines in rice. *OsActin* (*OsUBQ5*) was used as an internal control.

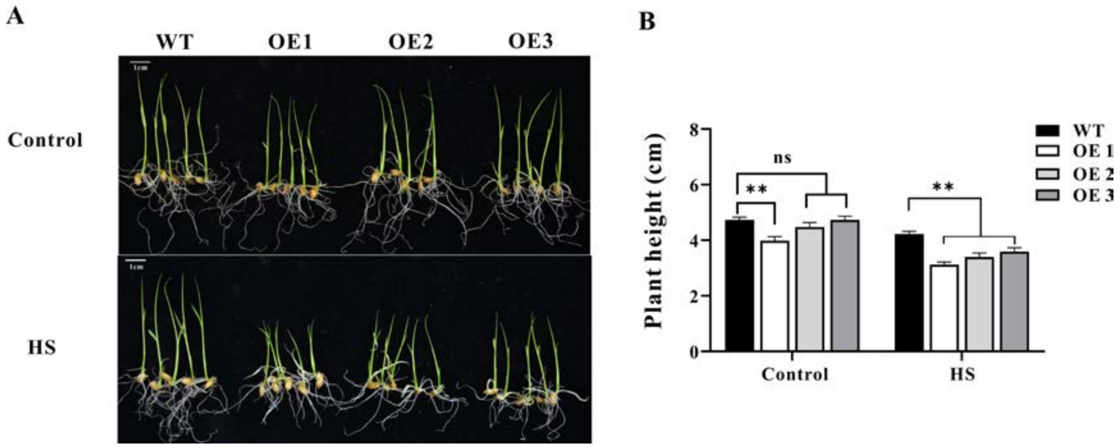

Figure S3. Enhanced heat sensitivity during germination in *ZmHsf11* transgenic rice. (A) Seed germination status of wild-type and transgenic plants after treatment at 35°C for 3 d and recovery at 28°C for 5 d. (B) Plant height measurements during germination period of wild-type and *ZmHsf11* transgenic seeds. Data are mean ± standard deviation (n = 15). **, P < 0.01.

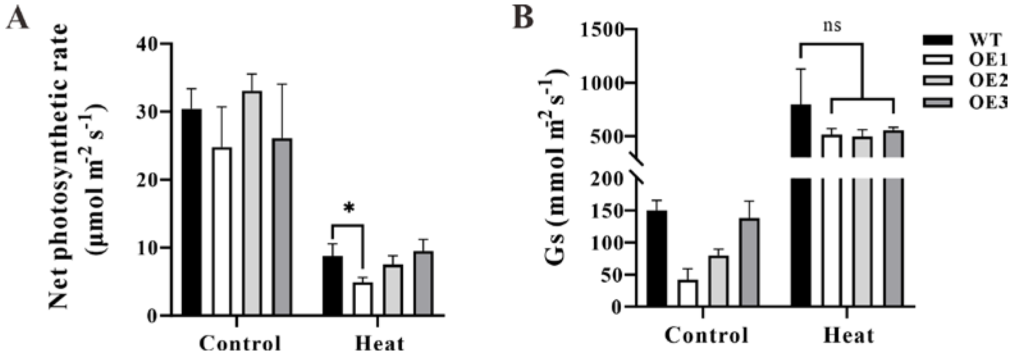

Figure S4. Physiological indicators of *ZmHsf11* transgenic and wild-type rice under heat stress. (A) Net photosynthetic rate. (B) Stomatal conductance. Data are means ± SDs of three independent sample replicates. *, P < 0.05.

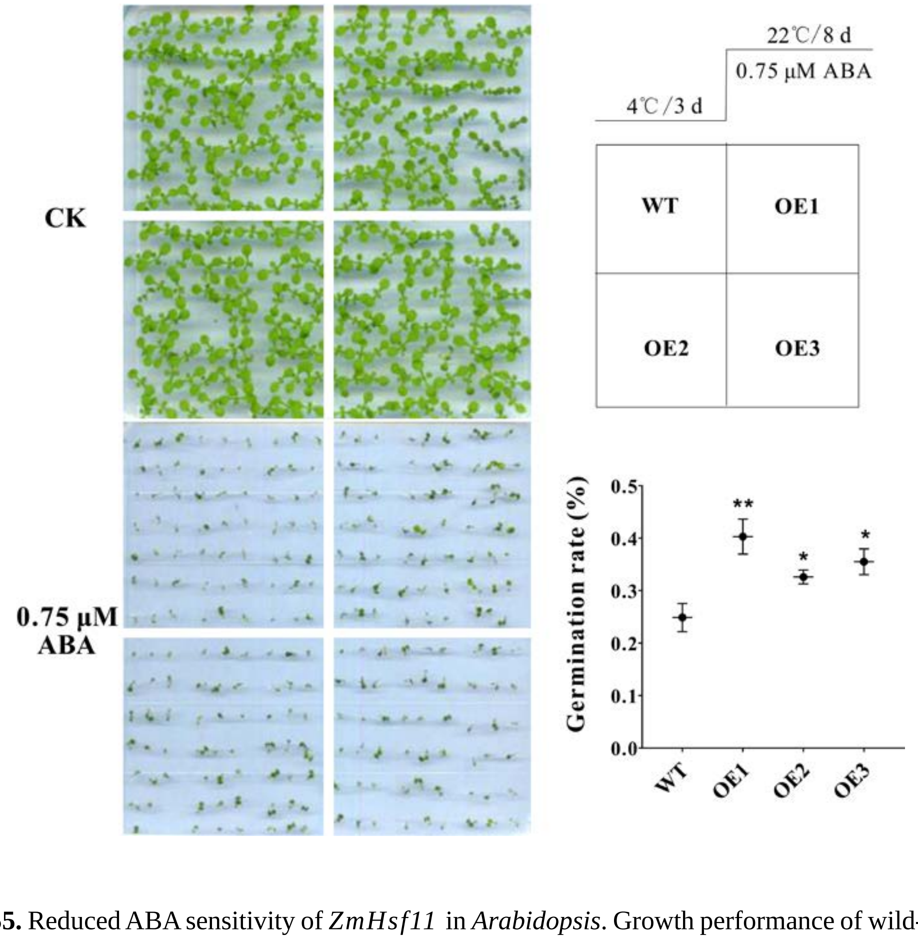

Figure S5. Reduced ABA sensitivity of *ZmHsf11* in *Arabidopsis*. Growth performance of wild-type and transgenic plants grown on MS medium containing 0.75 μM ABA concentrations after 8 d. Measurements of germination rate of wild-type and *ZmHsf11* transgenic seedlings. Data are means ± SDs (n = 36). *, P < 0.05; **, P < 0.01.

Table S1. Primer sequences used in this study.

Gene name	Forward primer (5'-3')	Reverse primer (5'-3')
ZmHsf11	ATGGCCGCCGAGCATGCCA	TCACCTCGAGTCGTTGGACCC
p1305-ZmHsf11	gctctagaATGGCCGCCGAGCATGCCA (Xba I)	cccccgggCCTCGAGTCGTTGGACCC (Sma I)
pGBKT7-ZmHsf11	atggccatggaggccgaattcATGGCCGCCGAGCATGCC	tcgacggatccccgggaattcTCACCTCGAGTCGTTGGAC
p1301a-ZmHsf11	gggtaccATGGCCGCCGAGCATGCCA (Kpn I)	gctctagaTCACCTCGAGTCGTTGGACCC (Pst I)
pCUB-ZmHsf11	ggatccccgggtaccgagctcATGGCCGCCGAGCATGCC	cgatcggggaaattcgagctcTCACCTCGAGTCGTTGGACC
qZmHsf11	CTGGGAGCGACCACGACG	AAACACTGGAGATTTTTACATAGG
ZmActin	GGGATTGCCGATCGTATGAG	GAGCCACCGATCCAGACACT
ZmGADPH	CTTCGGCATTGTTGAGGGTTTG	TCCTTGGCTGAGGGTCCGTC
AtRT-ZmHsf11	GGCTGCTCTGCGACATACA	ACTGCGGCTCCGTGGCGT
OsRT-ZmHsf11	ACGGGTTCAGGAAGATCGTG	ACGGGTTCAGGAAGATCGTG
AtUBQ5	AGGTTGAATCATCCGACA	GAGTCCACACTTACCACA
OsUBQ5	AGCAACTGGAAGATGGACG	CACCCCTCAGAGCAAGCAC
OsTUB8	CGCCGAAGGAGGAGAC	CACCTCCTCCTTGGCAG
OsGAPDH	TCGATGGCCCTTCAAT	TTCAATCCGGCAGGTC
OsDREB2A	TGAGATCCGTGAACCAA	CCATTAGCCACGATGAAA
OsHSFA2e	CTGAGTTCATACGCCAG	CATCTTGTTGCGCCTTG
OsNTL3	TCTGGCTTCTGCTGAT	CGACCACAGTTCTTCCT
OsHSP17.0	GTGTTCGACCCCTTCT	TCCTCCTTCTTGACGC
OsHSP18.0	CACCTGCTCGACATCC	GTACTTGCACGACTCCTC
OsGR	CCTCGAGTCCAAGATCAT	CCGTAGCCGCCTTCAC
OsAPX2	CTCCTACGCCGACTTCTA	TCTCCTTGTGGCATCTTC
OsRab7	AGCCGTGTGGTCTCTGAG	AATGGGTCCTTGAGAGTCAC
